# Norm-based comparison of the quality-of-life impact of ravulizumab and eculizumab in paroxysmal nocturnal hemoglobinuria

**DOI:** 10.1186/s13023-021-02016-8

**Published:** 2021-09-15

**Authors:** Carolyn E. Schwartz, Roland B. Stark, Katrina Borowiec, Sandra Nolte, Karl-Johan Myren

**Affiliations:** 1 DeltaQuest Foundation, Inc., 31 Mitchell Road, Concord, MA 01742 USA; 2 Departments of Medicine and Orthopaedic Surgery, Tufts University Medical School, Boston, MA USA; 3 Department of Measurement, Evaluation, Statistics, and Assessment, Boston College Lynch School of Education and Human Development, Chestnut Hill, MA USA; 4 Division of Psychosomatic Medicine, Medical Department, Charité – Universitätsmedizin Berlin, Corporate Member of Freie Universität Berlin and Humboldt-Universität Zu Berlin, Berlin, Germany; 5 Health Economics and Outcome Research, Alexion Pharmaceuticals, Inc., Stockholm, Sweden

**Keywords:** Quality of life, Paroxysmal nocturnal hemoglobinuria, Clinical trial, Response shift, Patient-reported outcome, EORTC, Norms, Eculizumab, Ravulizumab

## Abstract

**Aims:**

Paroxysmal nocturnal hemoglobinuria (PNH) is a rare and life-threatening intravascular hematologic disorder with significant morbidity and premature mortality. Clinical trials (NCT02946463 and NCT03056040) comparing ravulizumab with eculizumab for PNH have supported the non-inferiority of the former and similar safety and tolerability. This secondary analysis compared PNH trial participants after 26 weeks on either treatment (n = 438) to a general-population sample (GenPop) (n = 15,386) and investigated response-shift effects.

**Methods:**

Multivariate analysis of covariance (MANCOVA) investigated function and symptom scores on the European Organisation for Research and Treatment of Cancer (EORTC) QLQ-C30 of people with PNH as compared to GenPop, after covariate adjustment. Risk-factor groups were created based on clinical indicators known to be associated with worse PNH outcomes, and separate MANCOVAs were computed for lower- and higher-risk-factor groups. Differential item functioning (DIF) analyses examined whether item response varied systematically (1) by treatment, (2) compared to GenPop, and (3) over time, the latter two suggesting and reflecting response-shift effects, respectively. DIF analyses examined 24 items from scales with at least two items. *Recalibration response shift* was operationalized as uniform DIF over time, reflecting the idea that, for a given group, the difficulty of endorsing an item changes over time, after adjusting for the total subscale score. *Reprioritization response shift* was operationalized as non-uniform DIF over time, i.e., the relative difficulty of endorsing an item over time changes across the total domain score.

**Results:**

Across PNH risk-factor levels, people who had been on either treatment for 26 weeks reported better-than-expected functioning and lower symptom burden compared to GenPop. Ravulizumab generally showed larger effect sizes. Results were similar for lower and higher PNH risk factors, with slightly stronger effects in the former. DIF analyses revealed no treatment DIF, but did uncover group DIF (9 items with uniform DIF, and 11 with non-uniform) and DIF over time (7 items with uniform DIF, and 3 with non-uniform).

**Conclusions:**

This study revealed that people with PNH on ravulizumab or eculizumab for 26 weeks reported QOL levels better than those of the general population. Significant effects of DIF by group and DIF over time support recalibration and reprioritization response-shift effects. These findings suggest that the treatments enabled adaptive changes.

**Supplementary Information:**

The online version contains supplementary material available at 10.1186/s13023-021-02016-8.

## Introduction

Paroxysmal nocturnal hemoglobinuria (PNH) is a rare and life-threatening hematologic disorder with significant morbidity and premature mortality [1]. People with PNH may present with hemoglobinuria, thrombosis, impaired kidney function, abdominal pain, dysphagia, pulmonary hypertension, chest pain, dyspnea, erectile dysfunction in males, end organ damage, and/or severe fatigue [2–7]. PNH is characterized by dysregulation of the terminal complement pathway, leading to intravascular hemolysis and thrombosis. Such patients generally have a poor quality of life (QOL) [8]. If untreated, up to 35% die within 5 years of diagnosis [2, 3, 9–13]. Although onset can occur at any age, PNH has a worldwide mean age of diagnosis of 39.3 years (SD = 18.6) [2, 3, 14–16]. The prevalence rate is 12–13 per 1,000,000 persons and is similar across sexes but higher among older adults [17]. Its clinical course is highly unpredictable [3, 7]. Some patients have sudden onset and rapid progression to death, whereas others have long-term chronic illness but few life-threatening complications [3].

Eculizumab is a complement component-5 (C5) inhibitor that has been the standard of care since 2007, with evidence of lower mortality [18], improved QOL [19], reduced thrombosis risk, and normal life expectancy [9, 10, 12, 20]. Because of the treatment burden [21, 22] imposed by biweekly doses of eculizumab, recent clinical trials compared it with ravulizumab. Ravulizumab is a recently^1^ developed C5 inhibitor that produces immediate, complete, and sustained inhibition of C5 with an extended, 8-week dosing interval. Two head-to-head randomized clinical trials documented the non-inferiority, safety, tolerability and efficacy of the two drugs. Trial 301 (ALXN1210-PNH-301) [22] was implemented in people with PNH naïve to complement inhibitors [22]; Trial 302 (ALXN1210-PNH-302) [21], in people with PNH who were stable on eculizumab for at least 6 months and of which half were randomized to switch to ravulizumab [21]. The most frequently reported adverse event was headache, with slightly higher rates for ravulizumab [21].

One important indicator of treatment effectiveness is whether the treatment can enable a normal QOL; however, “normal” or near-normal levels is a “high bar” for conditions like PNH. It is a particularly challenging question because there is no validated disease-specific patient-reported outcome (PRO) measure for PNH [23]. Because PNH’s QOL impacts are similar to those of hematologic cancers, the pivotal trials collected data on cancer-specific QOL measures. Published results reported no difference between the treatments on the Functional Assessment of Chronic Illness (FACIT)-Fatigue [21, 22] and showed improvements on the European Organisation for Research and Treatment of Cancer (EORTC)—QLQ-C30 Global Health Status/QOL score [22]. Understanding how PNH EORTC scores compare to general-population values would be important for characterizing the QOL impact of ravulizumab and eculizumab.

A substantial evidence base of research across a broad range of patient populations has documented that people living with chronic or terminal illness evaluate their QOL differently than the general population does [24–42]. These response-shift effects reflect changes in their internal standards, values and/or conceptualization of QOL over time [43, 44]. Such changes might, for example, lead to a different way of thinking about “moderate” versus “little” fatigue compared to someone who has never had this blood disease (i.e., recalibration or change in internal standards). They may change their ideas of what is important to role functioning [45], for example, leading to different priorities and thus a different perspective on how well they are functioning (i.e., reprioritization or change in values) [40]. They may change the way they define QOL, for example by focusing less on economic or professional achievements and more on family welfare or intimacy (i.e., reconceptualization or change in conceptualization) [46]. Response-shift effects are natural and common concomitants to treatment outcomes [47–49]. When adaptive, they can help people maintain a homeostasis or stability in QOL that enables better affective and eudemonic well-being [50, 51].

We hypothesize that PNH patients whose condition is well-managed will evidence response-shift effects. Evaluating response-shift effects is akin to studying an iceberg: one notices the portion that stands out from the surface (e.g., surprising or paradoxical findings), and then examines indicators of what is below to characterize the object’s nature and size (e.g., information about differences in correlations among variables, item-response parameters, or cognitive-appraisal processes).

The present study thus evaluated the impact of ravulizumab and eculizumab on patients’ QOL as measured by the EORTC QLQ-C30 after 26 weeks of treatment, as compared to general-population norms. This treatment period is generally accepted as sufficient to achieve a stable, well-managed condition. The present work thus provides a normative comparison by examining the same PRO in people with PNH and the general population. The study then investigated response-shift effects by examining differential item functioning (DIF) [52]—by treatment, by group as compared to the general population, and over time, the latter two suggesting and reflecting response-shift effects.

## Methods

### Sample

This post-hoc secondary analysis utilized three data sources: two PNH clinical trials and one general-population study. Both trials were phase-3, open-label studies evaluating the non-inferiority of ravulizumab compared to eculizumab in changing primary and secondary clinical endpoints. Trial 301 (ALXN1210-PNH-301) was implemented in people with PNH not previously treated with complement inhibitors [22]; Trial 302 (ALXN1210-PNH-302), in people with PNH who were stable on eculizumab for at least 6 months and of whom half were randomized to switch to ravulizumab [21]. Data available for analysis included longitudinal follow-up from baseline through the extension trials, at which time all participants received ravulizumab, with total follow-up time typically 12 months (mean = 11.9; SD = 2.2; range = 0.3–19.4. For complete details on trial inclusion and exclusion criteria and procedures see references [21, 22]) The trial was conducted in accordance with the provision of the Declaration of Helsinki, the International Conference on Harmonization guidelines for Good Clinical Practice, and applicable regulatory requirements. The trial was approved by the institutional review board at each participating institution. All the patients provided written informed consent before participating.

The general-population study provided a 2015 cross-sectional sample from 11 European countries. Further country-specific norm data were obtained from Russia, Turkey, Canada, and the United States. Ethical approval was not sought as this study was solely based on panel research data collected by GfK SE. The survey conformed to the required ethical standards by obtaining written informed consent from all participants and collecting data completely anonymously [53].

### Measures

The EORTC QLQ-C30 is a comprehensive cancer-specific measure containing 30 items covering five function subscales (physical, role, emotional, cognitive, social); nine symptom subscales/items (fatigue, nausea/vomiting, pain, dyspnea, insomnia, appetite loss, constipation, diarrhea, financial difficulties); and a global health status/QOL subscale [54, 55]. Higher scores on the function and global health status/QOL scales and lower scores on the symptom scales reflect better health/QOL [56]. Of note, each individual item’s response options, except those for global health status/QOL, moved toward worsening health, which will be specifically relevant for selected analyses.

Demographic characteristics collected for all datasets included age, sex, and region. From the trial datasets, baseline clinical variables included in the analysis were lactate dehydrogenase or LDH stratum (< 1.5× upper limit of normal [ULN]; 1.5–< 3 × ULN; or ≥ 3 × ULN); pRBC stratum (0 units; 1–14; or > 14), and binary flags for aplastic anemia, immunosuppressant treatment, myelodysplastic syndrome, and bone marrow disorder.

### Statistical analysis

Analyses were conducted for the overall PNH group versus general population and by PNH risk-factor group. Risk-factor groups were created based on clinical indicators known to be associated with worse PNH outcomes (Table 1). An initial risk-factor score was based on a weighted sum of these indicators. The binary flags were given a weight of one (i.e., no = 1, yes = 2), whereas the LDH stratum was given a higher weight (i.e., stratum 1 = 2; stratum 2 = 4, stratum 3 = 6). Since pRBC was not used in the 302 trial, it was not included among the clinical indicators used for the risk-factor score. This weighting approach was based on input from a knowledgeable PNH clinician (AGK). The resulting score ranged from 6 to 12, and it was used to create a lower-risk-factor group (score 6–8) and a higher-risk-factor group (score 9–12).

**Table 1:** Deriving the PNH risk-factor score

Clinical indicator	Specific level of clinical indicator	Assigned value
Observed LDH category	LDH < 1.5×ULN	2
	LDH 1.5–< 3×ULN	4
	LDH ≥ 3xULN	6
Observed pRBC stratum	0 unit pRBC	NA
	1–14 units pRBC	NA
	> 14 units pRBC	NA
Immuno-suppressant treatment	No	1
	Yes	2
Aplastic anemia	No	1
	Yes	2
Myelodysplastic syndrome	No	1
	Yes	2
Bone marrow disorder	No	1
	Yes	2
*Summary score creation*		
Sum all assigned values	Sum assigned values for observed LDH, Immuno-suppressant treatment, aplastic anemia, myelodysplastic syndrome, and bone marrow disorder	
Create PNH risk-factor group		
	Lower	6–8
	Higher	9–12

NA: Since pRBC was not used in the 302 trial, it was not included among the clinical indicators used for the risk-factor score

Multivariate Analysis of Covariance (MANCOVA) compared people with PNH on ravulizumab or eculizumab after 26 weeks to the general-population sample. *Group* was coded such that those on ravulizumab and eculizumab were each compared to the general population, the referent group. Dependent variables for a first model included function and global-QOL scale scores, and for a second model, symptom scale scores/items. Age, sex, and region were included as covariates. MANCOVAs were also computed separately for lower and higher PNH-risk-factor groups as a way of adjusting for PNH severity.

Similar MANCOVA models were also computed by PNH-risk-factor group at baseline to check that results of the above models were likely results of treatment rather than of preexisting characteristics of the study samples.

Because the general-population sample was disproportionately large, model results are reported in terms of Cohen’s *d* statistic [57], expressed in standard-deviation units, to emphasize the degree to which group differences may have been clinically important. Using Cohen’s criteria, a *d* of 0.2–0.49 is considered a small effect size, 0.5–0.79 is medium, and 0.8 or greater is large [57].

Heat maps were used to illustrate group differences by computing this same effect size using means and standard deviations by age and gender groupings. Formatting of tables and figures illustrates effect-size magnitude, with more saturated color indicating larger effect.

Past research on item response and response shift have built on structural equation models [41] or item response theory (IRT) models [42]. Here, initial efforts used a bifactor model for the function scores (poor model fit) and multidimensional IRT models for function and symptom scores (models did not converge due to identifiability problems). The present work thus utilized a logistic-regression framework to test for DIF [43]. Accordingly, we adapted response-shift operationalizations by building upon this prior work.

In this study, *recalibration response shift* is operationalized as uniform DIF over time, because it reflects the idea that, for a given group, the difficulty of endorsing an item may change over time, after adjusting for the total subscale score (i.e., the latent trait). For example, uniform DIF would reflect a specific emotional-functioning item being easier or harder to endorse than one might expect, given a certain level of overall emotional functioning.

*Reprioritization response shift* is operationalized as non-uniform DIF over time because the relative difficulty of endorsing an item over time may change across the total score on the domain. This type of response shift is captured by item discrimination or slope. For example, non-uniform DIF would reflect a specific emotional-functioning item becoming easier or harder to endorse over time than one might expect, given a certain trajectory of overall emotional functioning.

DIF analyses [58, 59] were conducted on the 24 EORTC QLQ-C30 items belonging to scales with at least two items. The basic DIF analyses used ordinal logistic regression and involved building three nested models:

- *Model 1*: Logit[P(Y ≤ j)] = *α*_j_ + *b*_1_(Total Score);
- *Model 2*: Logit[P(Y ≤ j)] = *α*_j_ + *b*_1_(Total Score) + *b*_2_(Group); and
- *Model 3*: Logit[P(Y ≤ j)] = *α*_j_ + *b*_1_(Total Score) + *b*_2_(Group) + *b*_3_(Total Score * Group),

where P(Y ≤ j) represents the probability that *j* is the rating-scale response category, each *α*_j_ is a regression constant, and each *b* is a regression coefficient.^2^

The log-likelihood ratio test for statistical significance compared Model 1 versus 2, Model 2 versus 3, and Model 1 versus 3. *Uniform DIF* is characterized by b_2_ being significant and the log-likelihood test comparing Models 1 and 2 being significant (i.e., there is a significant main effect for Group). *Non-uniform DIF* is characterized by b_3_ being significant and the log-likelihood test comparing Models 2 and 3 being significant (i.e., there is a significant Group-by-total score interaction). Uniform *and* Non-uniform DIF is characterized by the log-likelihood test comparing Models 1 and 3 being significant.

DIF was computed in three ways to test distinct hypotheses, which tested one alternative explanation (first hypothesis) prior to testing for more definitive evidence of response-shift effects (second and third hypothesis, respectively):

*DIF by treatment* compared ravulizumab and eculizumab groups on item difficulty (threshold) and item discrimination (slope) in the longitudinal data. If significant, this type of DIF would suggest that the two treatment groups are responding differently to the EORTC items, and thus one cannot validly compare their responses.

*DIF by group* compared people with PNH to the general-population group at one point in time: after 26 weeks on therapy and at the single time point collected in the general-population study. In this analysis, domain scores were first grand-mean-centered to aid interpretation. When uniform DIF was detected, the associated odds ratio indicated the “favored” group: when > 1.0, the PNH group was more likely than expected to endorse (i.e., endorsing was “easier”); when < 1.0, the PNH group was less likely than expected to endorse (i.e., endorsing was “harder”). If the associated log-likelihood test’s *p* value was significant (i.e., < 0.05), this type of DIF showed that the groups were responding differently to the items. The use of the term “harder” reflects the centrality of the idea of *difficulty* in the study of item response. Greater item difficulty would mean a higher bar for endorsing a particular response option, given one’s total score on that domain. Such systematic differences between people with PNH and the general population would suggest that the two groups do not have a similar *contingent true score*, meaning that they are thinking about the QOL item(s) differently in terms of frame of reference, sampling of experience, standards of comparison, or patterns of emphasis. Fuller explanation of these concepts can be found in [46, 49]. Because the data testing this DIF hypothesis are measured at one point in time, response shift is not a definitive explanation and would require longitudinal data for confirmation.

*DIF over time* compared, for people with PNH, slopes and thresholds over the course of the pivotal and extension trials, to test for intra-individual changes. If significant, this type of DIF provides further support for recalibration and reprioritization response-shift effects. This DIF would demonstrate that individuals with PNH change the cognitive-appraisal processes underlying their item response, i.e., that their contingent true score changes over time.

Multilevel modeling was used to account for the multiple data points per person used for the DIF-by-treatment and DIF-over-time analyses. SPSS Release 27 [60] and Stata/IC 16.1 [61] were used for all analyses.

## Results

### Sample

The study samples included 441 people with PNH, of whom 246 had participated in trial 301 and 195 in trial 302. In trial 301, 214 people were on eculizumab and 224 on ravulizumab. In trial 302, 107 people were on eculizumab and 111 on ravulizumab. The PNH group was further characterized as 224 with lower and 217 higher levels of risk factors. The EORTC sample included 15,386 people. Table 2 provides descriptive statistics on demographic information shared between the two study samples. Table 3 provides clinical information about the PNH-treatment groups.

**Table 2:** Demographics of PNH patients at baseline compared to general population

Variable	Began trial on eculizumab (n = 219)		Began trial on ravulizumab (n = 222)		General population (n = 15,386)	
	Mean	SD	Mean	SD	Mean	SD
Age	47.38	15.30	45.67	14.83	53.57	15.375
Years Since Diagnosis	8.93	8.84	9.33	8.70	NA	
Baseline BMI (kg/m^2^)	25.25	4.23	24.83	4.71		

	#	%	#	%	#	%
Region						
Europe	113	51.60	106	47.75	13,373	86.92
Japan	22	10.05	25	11.26	0	0.00
Latin America	13	5.94	9	4.05	0	0.00
North America	16	7.31	17	7.66	2013	13.08
Rest of Asia Pacific	55	25.11	65	29.28	0	0.00
Female	102	46.58	107	48.20	7650	49.72

**Table 3:** Clinical characteristics of PNH patients at baseline

Variable	Began trial on eculizumab (n = 219)				Began trial on ravulizumab (n = 222)			
	Trial 301		Trial 302		Trial 301		Trial 302	
	#	%	#	%	#	%	#	%
Observed LDH Category								
LDH < 1.5×ULN	0	0	98	100	0	0	97	100
LDH 1.5–< 3×ULN	16	13	0	0	18	14	0	0
LDH ≥ 3×ULN	105	87	0	0	107	86	0	0
Observed pRBC stratum								
0 unit pRBC	21	17	NA		22	18	NA	
1–14 units pRBC	76	63			80	64		
> 14 units pRBC	24	20			23	18		
Immuno-suppressant treatment	14	12	98	100	15	12	97	100
Aplastic anemia	38	31	39	40	41	33	34	35
Myelodysplastic syndrome	6	5	6	6	7	6	3	3
Bone marrow disorder	43	36	42	43	46	37	35	36
Derived PNH risk-factor group								
Higher	107	88	0	0	110	88	0	0
Lower	14	12	98	100	15	12	97	100

	Mean	SD	Mean	SD	Mean	SD	Mean	SD
Derived PNH risk-factor index	9.21	0.93	6.46	0.56	9.22	0.87	6.38	0.53

### QOL comparison after 26 weeks

MANCOVA models revealed that across levels of PNH risk factors, patients who had been on either ravulizumab or eculizumab for 26 weeks reported better physical, emotional, and cognitive functioning, and lower nausea/vomiting, pain, insomnia, appetite loss, constipation, and diarrhea symptoms, than the general population, after adjusting for covariates (Table 4). Additionally, ravulizumab patients reported higher global health status/QOL, lower fatigue, and lower financial difficulties than the general population (Table 4). The effect sizes were generally larger for the ravulizumab patients.

**Table 4:**
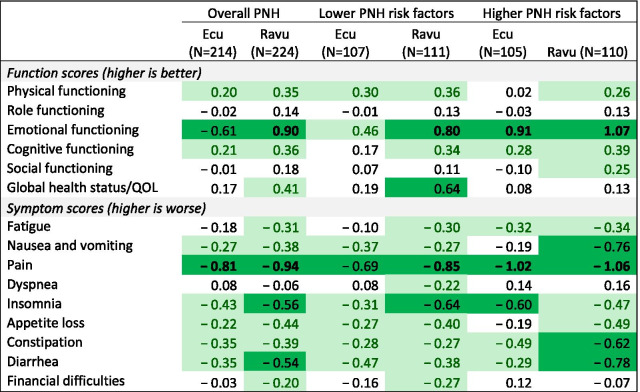
Effect sizes: PNH patients after 26 weeks of treatment compared to general population

Conditional formatting shows the magnitude and direction (green = better health status; red = worse health status) of the adjusted mean differences. General Population N = 15,386.

MANCOVA models conducted separately by risk level revealed further nuances in QOL after treatment. Similar to the overall MANCOVA, compared to the general population, both eculizumab and ravulizumab lower-risk-factor patients reported higher physical and emotional functioning and lower nausea/vomiting, pain, insomnia, appetite loss, constipation, and diarrhea symptoms. Further, the lower-risk-factor ravulizumab patients also reported better cognitive functioning and global QOL, and lower fatigue, dyspnea, and financial difficulties. In several domains, the effect sizes were larger for these ravulizumab patients (Table 4).

Models focused on the higher-risk-factor patients as compared to the general population revealed that people with PNH reported better emotional and cognitive functioning, and lower fatigue, pain, insomnia, constipation, and diarrhea (Table 4). Further, these ravulizumab patients also reported better physical and social functioning, and lower symptom burden in nausea/vomiting and appetite loss (Table 4). In almost all cases, these ravulizumab patients had larger effect sizes than the eculizumab patients (Table 4).

**Fig. 1:**
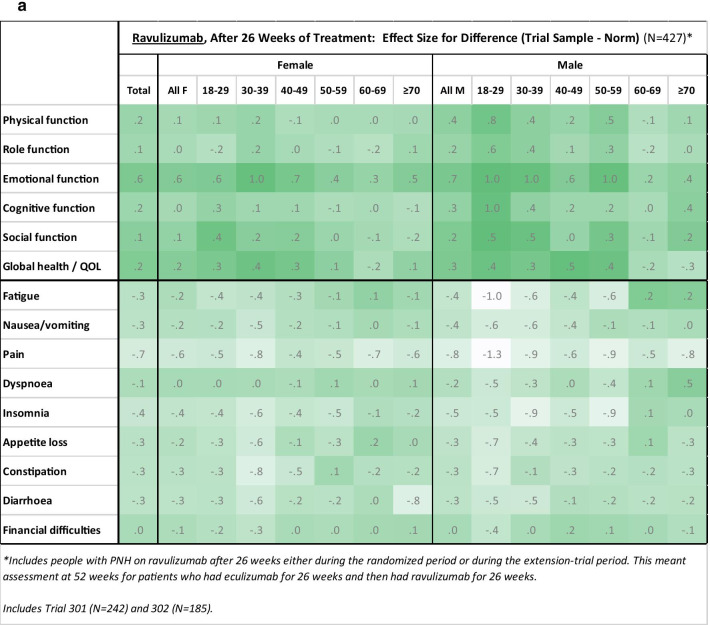

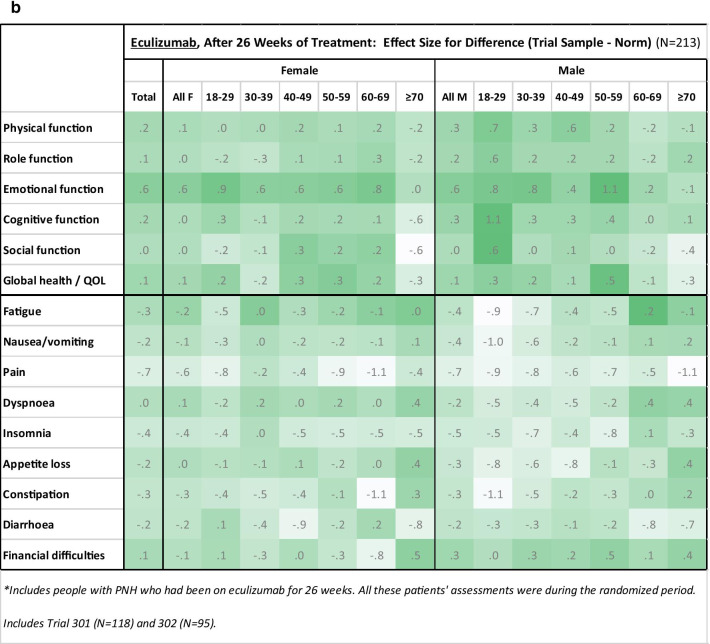
Heat maps. Heat maps illustrate group differences for ravulizumab (**a**) and eculizumab (**b**) using Cohen’s *d* effect size computed from aggregated means and standard deviations by age and gender groupings. Conditional formatting illustrates effect-size magnitude with a more saturated color reflecting larger effect size. Since all of the differences were in the direction of PNH group scoring better than the general population (i.e., higher on function/global QOL scales, lower on symptom scales/items), only one color is used for the conditional formatting. Figure **a** includes people with PNH on ravulizumab after 26 weeks either during the randomized period or during the extension-trial period. This meant assessment at 52 weeks for patients who had eculizumab for 26 weeks and then had ravulizumab for 26 weeks. Includes Trial 301 (N = 242) and 302 (N = 185). Figure **b** includes people with PNH who had been on eculizumab for 26 weeks. All these patients' assessments were made during the randomized period. Includes Trial 301 (N = 118) and 302 (N = 95)

Figure 1a and b show heat maps comparing treated patients to general-population norms. Since all of the differences showed better scores for the PNH group (i.e., higher on function/global QOL scales, lower on symptom scales/items), only one color is used for the conditional formatting. These graphs suggest that generally the effects were larger for the function scales than for the symptom scales/items and larger for ravulizumab patients than for eculizumab patients.

### QOL comparison at baseline

Because many of these findings were counter to expectation (i.e., functioning and symptom scores that were *better* than in the general population), we implemented similar MANCOVAs using the baseline data of trial patients who were treatment-naïve (from trial 301), to check whether the results were more likely due to treatment or to stable participant characteristics. These sample sizes are substantially smaller due to excluding patients from trial 302 while also splitting the analysis by level of risk. Results show that in general and as expected, untreated people with PNH at baseline reported worse function and symptom scores than did the general population. The exceptions generally involved small effects. (Additional file 1: Table S1).

### DIF by treatment

Results of multilevel DIF analysis by treatment group revealed no significant effects in any of the 24 EORTC QLQ-C30 items (Table 5). Thus, across the multiple time points, there is no indication of treatment-related DIF, and one can compare responses of people with PNH regardless of the treatment they have received. In other words, given the same total score, people in the two groups responded similarly to a given item in that scale.

**Table 5:** Results of DIF analyses by treatment group

Item	Label	Domain	DIF by treatment group			
			Test of uniform DIF		Test of non-uniform DIF	
			*p* value (on group effect)	“Favored”* group	Odds ratio on group effect	*p* value (on interaction term)
eortc29	29. How would you rate your overall health during the past week?	Global	NS	NS	NS	NS
eortc30	30. How would you rate your overall quality of life during the past week?	Global	NS	NS	NS	NS
eortc01	1. Do you have any trouble doing strenuous activities, like carrying a heavy shopping bag or a suitcase?	Physical	NS	NS	NS	NS
eortc02	2. Do you have any trouble taking a long walk?	Physical	NS	NS	NS	NS
eortc03	3. Do you have any trouble taking a short walk outside of the house?	Physical	NS	NS	NS	NS
eortc04	4. Do you need to stay in bed or a chair during the day?	Physical	NS	NS	NS	NS
eortc05	5. Do you need help with eating, dressing, washing yourself or using the toilet?	Physical	NS	NS	NS	NS
eortc06	6. Were you limited in doing either your work or other daily activities?	Role	NS	NS	NS	NS
eortc07	7. Were you limited in pursuing your hobbies or other leisure time activities?	Role	NS	NS	NS	NS
eortc21	21. Did you feel tense?	Emotional	NS	NS	NS	NS
eortc22	22. Did you worry?	Emotional	NS	NS	NS	NS
eortc23	23. Did you feel irritable?	Emotional	NS	NS	NS	NS
eortc24	24. Did you feel depressed?	Emotional	NS	NS	NS	NS
eortc20	20. Have you had difficulty in concentrating on things, like reading a newspaper or watching television?	Cognitive	NS	NS	NS	NS
eortc25	25. Have you had difficulty remembering things?	Cognitive	NS	NS	NS	NS
eortc26	26. Has your physical condition or medical treatment interfered with your family life?	Social	NS	NS	NS	NS
eortc27	27. Has your physical condition or medical treatment interfered with your social activities?	Social	NS	NS	NS	NS
eortc10	10. Did you need to rest?	Fatigue	NS	NS	NS	NS
eortc12	12. Have you felt weak?	Fatigue	NS	NS	NS	NS
eortc18	18. Were you tired?	Fatigue	NS	NS	NS	NS
eortc14	14. Have you felt nauseated?	Nauseau	NS	NS	NS	NS
eortc15	15. Have you vomited?	Nauseau	NS	NS	NS	NS
eortc09	9. Have you had pain?	Pain	NS	NS	NS	NS
eortc19	19. Did pain interfere with your daily activities?	Pain	NS	NS	NS	NS

*"Favored" = Finds it easier to endorse poor health except for eortc29 and eortc30

### DIF by group

Results of PNH versus general-population groups’ DIF analysis revealed uniform DIF in 14 items (Table 6). Most often it was more difficult for the treatment group to report that they had poor health. This was true for 9 of these items (1 physical item, 2 emotional, 1 cognitive, 2 social, 1 fatigue, 1 nausea, and 1 pain). In 5 of these items (1 physical, 1 emotional, 1 cognitive, 1 fatigue, 1 nausea), it was more difficult for the general-population group to report poorer health.

**Table 6:** Results of DIF analyses of PNH versus general population

Item	Label	Domain	DIF by Group (PNH vs. general population)					
			Test of uniform DIF			Test of non-uniform DIF		
			Likelihood ratio test *p* value	"Favored" group	Odds ratio on group effect	Likelihood ratio test *p* value	"Favored" group at the mean total domain score	Odds ratio on group effect at the mean total domain score
eortc29	29. How would you rate your overall health during the past week?	Global	NS	NS	NS	NS	NS	NS
eortc30	30. How would you rate your overall quality of life during the past week?	Global	NS	NS	NS	NS	NS	NS
eortc01	1. Do you have any trouble doing strenuous activities, like carrying a heavy shopping bag or a suitcase?	Physical	*p* < .001	General pop	0.5326	*p* = .0214	General pop	0.5599
eortc02	2. Do you have any trouble taking a long walk?	Physical	*p* < .001	Ecu/Ravu	1.9937	NS	NS	NS
eortc03	3. Do you have any trouble taking a short walk outside of the house?	Physical	NS	NS	NS	NS	NS	NS
eortc04	4. Do you need to stay in bed or a chair during the day?	Physical	NS	NS	NS	*p* = .0089	Ecu/Ravu	1.0833
eortc05	5. Do you need help with eating, dressing, washing yourself or using the toilet?	Physical	NS	NS	NS	NS	NS	NS
eortc06	6. Were you limited in doing either your work or other daily activities?	Role	NS	NS	NS	NS	NS	NS
eortc07	7. Were you limited in pursuing your hobbies or other leisure time activities?	Role	NS	NS	NS	NS	NS	NS
eortc21	21. Did you feel tense?	Emotional	*p* < .001	General pop	0.2567	NS	NS	NS
eortc22	22. Did you worry?	Emotional	NS	NS	NS	*p* < .001	Ecu/Ravu	1.3910
eortc23	23. Did you feel irritable?	Emotional	*p* < .001	Ecu/Ravu	2.8577	NS	NS	NS
eortc24	24. Did you feel depressed?	Emotional	*p* < .001	General pop	0.5886	*p* < .001	General pop	0.7189
eortc20	20. Have you had difficulty in concentrating on things, like reading a newspaper or watching television?	Cognitive	*p* < .001	General pop	0.2165	*p* < .001	General pop	0.1177
eortc25	25. Have you had difficulty remembering things?	Cognitive	*p* < .001	Ecu/Ravu	1.9155	*p* < .001	Ecu/Ravu	1.6161
eortc26	26. Has your physical condition or medical treatment interfered with your family life?	Social	*p* < .001	General pop	0.3198	*p* = .0003	General pop	0.5326
eortc27	27. Has your physical condition or medical treatment interfered with your social activities?	Social	*p* < .001	General pop	0.3135	*p* < .001	General pop	0.1755
eortc10	10. Did you need to rest?	Fatigue	*p* < .001	General pop	0.3642	NS	NS	NS
eortc12	12. Have you felt weak?	Fatigue	NS	NS	NS	NS	NS	NS
eortc18	18. Were you tired?	Fatigue	*p* < .001	Ecu/Ravu	2.4351	*p* < .001	Ecu/Ravu	2.4843
eortc14	14. Have you felt nauseated?	Nauseau	*p* = .0253	Ecu/Ravu	1.8589	*p* < .001	General pop	0.4916
eortc15	15. Have you vomited?	Nauseau	*p* = .0006	General pop	0.1791	NS	NS	NS
eortc09	9. Have you had pain?	Pain	*p* < .001	General pop	0.3012	NS	NS	NS
eortc19	19. Did pain interfere with your daily activities?	Pain	NS	NS	NS	*p* < .001	Ecu/Ravu	1.4191

*"Favored" = Finds it easier to endorse poor health except for eortc29 and eortc30

Non-uniform DIF was detected in 11 items, 6 favoring the general population at the domain score mean, meaning that it was easier for them to report poorer health (1 physical, 1 emotional, 1 cognitive, 2 social, 1 nausea), suggesting that this group effect varied by level of the EORTC QLQ-C30 item. There were 5 items favoring the PNH group, meaning that it was easier for them to report poorer health (1 physical, 1 emotional, 1 cognitive, 1 fatigue, and 1 pain).

### DIF over time

Results of multilevel DIF analysis evaluating the impact of time on people with PNH item responses revealed significant uniform DIF effects in 7 of the 24 items (Table 7). These differences related to physical function (2 of 5 items), role function (2 of 2), emotional function (1 of 4), fatigue (1 of 3), and pain (1 of 2). These DIF effects suggested a decreasing likelihood over time of endorsing physical function problems, fatigue, and pain symptoms, given their total scores on the corresponding scales. In contrast, there was an increasing likelihood of endorsing irritability (emotional function item). For the two role-function items, one result showed an increase and one a decrease, thereby canceling each other out. Three of 24 items showed evidence of non-uniform DIF: 1 emotional, 1 fatigue, and 1 pain. Thus, there is evidence of recalibration response-shift effects in 7 of 24 items, and reprioritization response-shift effects in 3 items.

**Table 7:** Results of DIF analyses over time

Item	Label	Domain	DIF over time within PNH patients			
			Test of uniform DIF		Test of non-uniform DIF	
			*p* value (on group effect)	"Favored" group	Odds ratio on group effect^φ^	*p* value (on interaction term)
eortc29	29. How would you rate your overall health during the past week?	Global	NS	NS	NS	NS
eortc30	30. How would you rate your overall quality of life during the past week?	Global	NS	NS	NS	NS
eortc01	1. Do you have any trouble doing strenuous activities, like carrying a heavy shopping bag or a suitcase?	Physical	*p* = .001	As time increases, the likelihood of endorsing **decreases**	0.94	NS
eortc02	2. Do you have any trouble taking a long walk?	Physical	*p* = .008	As time increases, the likelihood of endorsing **decreases**	0.95	NS
eortc03	3. Do you have any trouble taking a short walk outside of the house?	Physical	NS	NS	NS	NS
eortc04	4. Do you need to stay in bed or a chair during the day?	Physical	NS	NS	NS	NS
eortc05	5. Do you need help with eating, dressing, washing yourself or using the toilet?	Physical	NS	NS	NS	NS
eortc06	6. Were you limited in doing either your work or other daily activities?	Role	*p* < .001	As time increases, the likelihood of endorsing **increases**	1.08	NS
eortc07	7. Were you limited in pursuing your hobbies or other leisure time activities?	Role	*p* < .001	As time increases, the likelihood of endorsing **decreases**	0.90	NS
eortc21	21. Did you feel tense?	Emotional	NS	NS	NS	NS
eortc22	22. Did you worry?	Emotional	NS	NS	NS	NS
eortc23	23. Did you feel irritable?	Emotional	*p* = .009	As time increases, the likelihood of endorsing **increases**	1.04	*p* = .031
eortc24	24. Did you feel depressed?	Emotional	NS	NS	NS	NS
eortc20	20. Have you had difficulty in concentrating on things, like reading a newspaper or watching television?	Cognitive	NS	NS	NS	NS
eortc25	25. Have you had difficulty remembering things?	Cognitive	NS	NS	NS	NS
eortc26	26. Has your physical condition or medical treatment interfered with your family life?	Social	NS	NS	NS	NS
eortc27	27. Has your physical condition or medical treatment interfered with your social activities?	Social	NS	NS	NS	NS
eortc10	10. Did you need to rest?	Fatigue	NS	NS	NS	NS
eortc12	12. Have you felt weak?	Fatigue	*p* < .001	As time increases, the likelihood of endorsing **decreases**	0.93	NS
eortc18	18. Were you tired?	Fatigue	NS	NS	NS	*p* = .003
eortc14	14. Have you felt nauseated?	Nauseau	NS	NS	NS	NS
eortc15	15. Have you vomited?	Nauseau	NS	NS	NS	NS
eortc09	9. Have you had pain?	Pain	NS	NS	NS	*p* = .002
eortc19	19. Did pain interfere with your daily activities?	Pain	*p* = .047	As time increases, the likelihood of endorsing **decreases**	0.95	NS

*"Favored" = Finds it easier to endorse poor health except for eortc29 and eortc30

^φ^Odds ratio converted to by-month estimate for ease of interpretation (i.e., daily estimate^30)

## Discussion

This study revealed that people with PNH on eculizumab and especially ravulizumab for 26 weeks reported QOL levels better than those of the general population, typically by 0.3 standard deviations. Not only was ravulizumab not inferior to eculizumab [21, 22], but both treatments also appeared to make QOL with PNH at least as good as the norm. These findings were equally notable for lower- and higher-risk-factor patients. In contrast, at baseline and prior to treatment, people with PNH,^3^ especially those categorized with higher-risk-factor PNH, were generally worse off than the general population.

DIF analyses revealed group- and time-related DIF, but not treatment-related DIF. Thus, there were no systematic differences in item response between these two effective PNH treatments, but there were in analyses comparing people with PNH to the general population, and to themselves over time. Specifically, compared to the general population, people with PNH after 26 weeks of effective treatment tended to be less likely than expected to endorse poor health. For example, they were less likely to endorse having trouble concentrating than one might expect given their overall level of cognitive function (uniform DIF or recalibration). This effect for concentration was even more pronounced over levels of the trait (non-uniform DIF or reprioritization).

These recalibration and reprioritization effects reflect adaptive response shifts. In this way, the scores of people with PNH, irrespective of treatment, not only approached “normal” QOL, but even “better than normal.” This pattern of responses suggests that ravulizumab and eculizumab enabled patients not only to achieve a better QOL but also to adapt to their condition. For example, they may have been aware of being fatigued while at the same time noting that it was less debilitating than it used to be. Thus, compared to the general population, the same level of feeling heavy and lethargic may have been calibrated as less onerous for someone with PNH. This recalibration response shift would continue over time, making their earlier and later responses less-than-comparable because of differences in their contingent true score (e.g., comparing their QOL to different standards). As another related dynamic, they may have modified their daily responsibilities or hobbies, so that the activities were more feasible. In this new context, it would be more difficult for them to report that these activities were limited by their condition (reprioritization response shift).

PNH is a difficult disease to live with. Its many signs and symptoms involve multiple organ systems, and the uncertainty that people with PNH experience makes these function- and symptom-impacts even more challenging. A treatment that provides immediate, complete and sustained C5 inhibition not only brings QOL to a normal level, but it enables adaptation, which may have an even greater value. For someone who knows what debilitating fatigue is, being given the opportunity to experience life without fatigue makes those days all the more poignant and joyful.

The present work had many strengths, including robust sample sizes and the use of a general-population comparison sample. Its limitations must, however, be acknowledged. First, the comparison group was very large at 15,000, and so the multivariable analyses had sufficient power to detect very small effect sizes. This hypersensitivity is why we emphasize Cohen’s *d* effect sizes. Caution is also warranted in interpreting results because of the few items in each scale, especially when there are only two. Future research might replicate the response-shift analyses on groups of more similar size, or might investigate the longitudinal-DIF findings using measures of QOL cognitive appraisal [29] or interviews. Given the rarity of PNH, this replication would be challenging. Finally, in the multivariate analyses comparing people with PNH and the general population, we were ultimately able to adjust only for age, sex, and region. Other variables unexamined and unavailable in this study might be relevant to explaining or mediating these group differences, such as expectations.

In summary, people with PNH who were treated for 26 weeks with eculizumab or ravulizumab not only showed comparable effects on clinical outcomes, but also showed a notable and important QOL benefit—especially with ravulizumab. People with PNH also provided evidence of response shifts over time, suggesting that the treatments enabled adaptive changes.

## Supplementary Information

**Additional file 1: Table S1.** Baseline and treatment-naive PNH comparisons to general population (Cohen’s d).

## Data Availability

Alexion will consider requests for disclosure of clinical study participant-level data provided that participant privacy is assured through methods like data de-identification, pseudonymization, or anonymization (as required by applicable law), and if such disclosure was included in the relevant study informed consent form or similar documentation. Qualified academic investigators may request participant-level clinical data and supporting documents (statistical analysis plan and protocol) pertaining to Alexion-sponsored studies. Further details regarding data availability and instructions for requesting information are available in the Alexion Clinical Trials Disclosure and Transparency Policy at https://alexion.com/our-research/research-and-development. The EORTC norm data may be requested via https://www.eortc.org/data-sharing/.

## Abbreviations

C5: Component-5
DIF: Differential item functioning
EORTC QLQ-C30: European Organisation for Research and Treatment of Cancer Quality of Life Questionnaire-C30
MANCOVA: Multivariate analysis of covariance
PNH: Paroxysmal nocturnal hemoglobinuria
PRO: Patient-reported outcome
QOL: Quality of life
SD: Standard deviation

## Footnotes

1 FDA Dec 2018, EMA July 2019.

2 Technical note: The standard ordinal logistic regression model parameterization indicates the probability of moving to a lower category for each unit increase in *X*. Stata transforms the output such that positive coefficients indicate higher probabilities of moving to a “higher” category for each unit increase in *X*, easing interpretation.

3 In this analysis we only included patients from trial 301 as these patients were untreated at baseline, whereas patients in trial 302 were stable at trial entry.
