# Supplementary material for: Norm-based comparison of the quality-of-life impact of ravulizumab and eculizumab in paroxysmal nocturnal hemoglobinuria
Source: Orphanet J Rare Dis. 2021 Sep 15;16:389. doi: 10.1186/s13023-021-02016-8 (PMC8442345; doi:10.1186/s13023-021-02016-8)
Supplement: Supplementary file 1 — Additional file 1: Table S1. Baseline and treatment-naive PNH comparisons to general population (Cohen’s d). [file 13023_2021_2016_MOESM1_ESM.pdf]

Supplemental Table 1. Baseline and treatment-naive PNH Comparisons to General Population (Cohen's *d*)*				
	Lower PNH Risk Factors		Higher PNH Risk Factors	
	Ecu (N=42)	Ravu (N=14)	Ecu (N=106)	Ravu (N=111)
**Function Scores *(higher is better)***				
Physical Functioning	-0.23	-0.62	-0.65	-0.70
Role Functioning	-0.18	-0.74	**-0.82**	-0.78
Emotional Functioning	0.02	-0.31	-0.28	-0.25
Cognitive Functioning	0.35	-0.19	-0.49	-0.43
Social Functioning	-0.25	-0.77	-0.69	-0.64
Global Health Status/QOL	-0.23	-0.73	-0.64	-0.51
**Symptom Scores *(higher is worse)***				
Fatigue	0.50	**1.03**	0.65	0.76
Nausea and Vomiting	-0.26	0.79	0.07	0.06
Pain	-0.30	0.39	-0.09	0.00
Dyspnoea	0.66	0.46	**1.06**	**0.96**
Insomnia	-0.45	0.14	0.03	0.10
Appetite Loss	-0.17	0.65	0.49	0.34
Constipation	-0.42	-0.28	-0.19	-0.31
Diarrhea	0.01	0.20	-0.22	-0.28
Financial Difficulties	-0.12	0.23	0.57	0.52

** Each d statistic represents (PNH group - General Population group)/pooled SD.*

Conditional formatting shows the magnitude and direction (green = better health status; red = worse healthstatus) of the correlations.

General Population N=15,386.
